# Neurorehabilitation Combining Acupuncture and Conventional Rehabilitation in the Acute Ischemic Stroke: Evidence From Multimodal MRI

**DOI:** 10.1155/np/8734736

**Published:** 2026-06-22

**Authors:** Feng Li, Jing Wang, Qilan Xu, Zhengyang Zhu, Mo Cheng, Jiu Chen, Bing Zhang

**Affiliations:** ^1^ Department of Neurology, Lu’an Hospital of Anhui Medical University, Lu’an People’s Hospital of Anhui Province, Lu’an, Anhui, China; ^2^ Graduate School of Bengbu Medical College, Bengbu, Anhui, China; ^3^ Department of Radiology, Lu’an Hospital of Anhui Medical University, Lu’an People’s Hospital of Anhui Province, Lu’an, Anhui, China; ^4^ Medical Imaging Center, Affiliated Drum Tower Hospital, Medical School of Nanjing University, Nanjing, Jiangsu, China, nju.edu.cn; ^5^ Lu’an Hospital of Anhui Medical University, Lu’an People’s Hospital of Anhui Province, Lu’an, Anhui, China; ^6^ Institute of Medical Imaging and Artificial Intelligence, Nanjing University, Nanjing, Jiangsu, China, nju.edu.cn; ^7^ Department of Radiology, The Affiliated Drum Tower Hospital of Nanjing University Medical School, Nanjing, Jiangsu, China, nju.edu.cn; ^8^ Jiangsu Key Laboratory of Molecular Medicine, Nanjing, Jiangsu, China; ^9^ Institute of Brain Science, Nanjing University, Nanjing, Jiangsu, China, nju.edu.cn

**Keywords:** acupuncture, acute ischemic stroke, functional connectivity, gray matter volume

## Abstract

**Background:**

Acute ischemic stroke (AIS) remains a leading cause of long‐term disability, with approximately 70% of survivors suffering from motor, sensory, and language impairments that significantly affect their daily functioning and quality of life. Recent advances in neuroimaging have provided a more precise assessment of changes in brain gray matter volume (GMV) and functional connectivity (FC), both of which are closely correlated with disease severity, prognosis, and rehabilitation outcomes. Acupuncture, as a complementary therapy, has demonstrated potential in alleviating motor and language deficits following stroke. By integrating structural magnetic resonance imaging (sMRI) and functional MRI (fMRI) data with comprehensive clinical evaluations, this research aims to objectively investigate the neural mechanisms underlying acupuncture‐induced brain plasticity. Our goal is to explore how acupuncture may augment conventional AIS treatments by promoting neuroplasticity, offering a scientific basis for its integration into standard therapeutic protocols. The findings aim to advance our understanding of the neurobiological basis for integrative rehabilitation strategies, ultimately contributing to improved prognostic evaluation and optimized recovery trajectories for AIS patients.

**Methods:**

A total of 54 AIS patients with motor dysfunction were allocated to receive either acupuncture combined with western medicine treatment (AWT) group or WT group alone. The acupuncture points used included: Neiguan (PC6), Renzhong (DU26), Sanyinjiao (SP6), Weizhong (BL40), Zusanli (ST36), Fenglong (ST40), Taichong (LR3), and Yanglingquan (GB34). Patients in AWT groups received treatment 5 times per week for 8 weeks. National Institutes of Health Stroke Scale (NIHSS), the Fugl‐Meyer assessment (FMA) and structural and fMRI data were collected at three time points: baseline (on the day of enrollment), week 8 (at the end of the acupuncture intervention), and week 12 (after a 12‐week follow‐up period post‐intervention). GMV and FC analysis was performed to investigate the potential mechanism of acupuncture treatment by comparing differences in brain cortical structure and function between treatments.

**Results:**

GMV: The AWT group manifested an increased GMV in the ipsilesional supplementary motor area (SMA) and contralesional anterior cingulate gyrus (ACG) by week 8. In contrast, the WT group witnessed a substantial decline in GMV within the ipsilesional median cingulate and paracingulate gyrus (DCG). At the 12‐week follow‐up, the AWT group further demonstrated a significantly greater increase in GMV in the ipsilesional superior temporal gyrus (STG) when compared to week 8. The between‐group comparisons at week 8 disclosed that the AWT group had a significantly elevated GMV in the postcentral gyrus (PoCG) and contralesional inferior temporal gyrus (ITG) (*p*  = 0.005) in contrast to the WT group. By week 12, the AWT group also presented a marked increase in GMV in the ipsilesional ITG relative to the WT group. In the spearman correlation analysis, a positive correlation was identified in the AWT group between the GMV of the ipsilesional SMA at 8 weeks post‐enrollment and the FMA score at week 12 (*p*  = 0.016, *R* = 0.487). Moreover, the GMV in the contralesional ACG exhibited a positive correlation with the FMA score at week 12 (*p* = 0.006, *R* = 0.540). Additionally, a negative correlation was also detected in the WT group between the GMV of contralesional PoCG and the NIHSS score at week 8 (*p* = 0.004, *R* = −0.540). FC: At 8 weeks post‐enrollment, the AWT group exhibited significantly increased FC between the ipsilesional SMA and the contralesional middle temporal gyrus (MTG), between the contralesional PoCG and the ACG, and between the contralesional PoCG and the ipsilesional pallidum (PAL). In the WT group, increased FC was observed between the ipsilesional SMA and the contralesional STG, and between the contralesional PoCG and the ipsilesional middle occipital gyrus (MOG). No significant positive FC regions were identified in the between‐group comparisons at either 8 weeks post‐enrollment or the 12‐week follow‐up. In the spearman correlation analysis, the enhanced FC between the ipsilesional SMA and the contralesional MTG in the AWT group was positively correlated with the post‐acupuncture FMA score (*p* = 0.043, *R* = 0.417). In the WT group, the enhanced FC between the contralesional PoCG and the ipsilesional MOG was positively correlated with the FMA score (*p* = 0.003, *R* = 0.567).

**Conclusions:**

The increased in GMV and enhanced FC in AIS patients following acupuncture intervention are correlated with improvements in neurological and motor functions. Acupuncture may promote neural network remodeling and the coordinate of brain structure and function. These findings suggest potential neurobiological mechanisms through which acupuncture can improve clinical outcomes in patients.

## 1. Introduction

Stroke is the second leading cause of disability and death worldwide [1]. Approximately 75% of stroke survivors experience sequelae, with 40% having severe disabilities, including limb movement, language, and cognitive disorders [2]. With advancements in neuroimaging techniques, the brain’s ability to undergo self‐repair can be detected and assessed through changes in brain structure and function [3]. Although the brain can undergo self‐repair to some extent by undergoing structural and functional changes, this level of repair is insufficient for stroke patients; therefore, external stimulus‐based interventions are still required. Therefore, this study focused on the structural and functional remodeling of the cerebral cortex during treatment with acupuncture for acute ischemic stroke (AIS).

Acupuncture is widely acknowledged as an effective treatment for stroke rehabilitation, having been used for thousands of years to enhance motor, sensation, and various neurological functions post‐stroke [4–10]. Acupuncture can improve patients’ Barthel index (BI) and their activities of daily living (ADL). Specific acupuncture points, such as Zusanli (ST36), Hegui (LI4), and Sanyingjiao (SP6), can also improve neurological function through different pathways [11, 12], while Neiguan (PC6) can enhance neurological function, reduce cerebral infarction volume, and extend the time window for AIS rats [13]. However, much less is known about the neuroimaging mechanism of acupuncture in the therapeutic rehabilitation of AIS patients.

This study aims to explore alterations in cortical structure and function following acupuncture intervention. We evaluated the clinical effects of acupuncture on motor dysfunction in patients with AIS at multiple time points, and investigated its central mechanisms using structural magnetic resonance imaging (sMRI) and resting‐state functional MRI (rs‐fMRI), focusing on gray matter volume (GMV) and functional connectivity (FC). The findings of this research are expected to provide valuable insights into the clinical efficacy and neural mechanisms underlying acupuncture treatment for stroke patients.

## 2. Materials and Methods

### 2.1. Participants

Patients with AIS who presented at Lu’an People’s Hospital of Anhui Province from October 2021 to October 2023 were enrolled in this study. A total of 54 AIS patients included in the baseline period were randomized using SPSS 26.0 statistical software for clinical trials. Participants were randomly categorized into two groups: the western medicine treatment (WT) group and the acupuncture combined with WT (AWT) group. The acupuncture points used included: Neiguan (PC6), Renzhong (DU26), Sanyinjiao (SP6), Weizhong (BL40), Zusanli (ST36), Fenglong (ST40), Taichong (LR3), and Yanglingquan (GB34). All acupoints were located according to the national standard of the People’s Republic of China (2006), Names and Locations of Acupoints (GB/T12346‐2006). Each AIS patient received acupuncture for 30 min per session, five times a week, for a course of 8 weeks. All acupuncture procedures were conducted by the same experienced and licensed acupuncturist. With the approval of the hospital ethics committee (2021LL021), each participant signs an informed consent form after full understanding. All patients received routine conventional rehabilitation during the 3‐month study period. The frequency, duration, and intensity of conventional rehabilitation training were comparable between the two groups throughout the intervention period.

### 2.2. Inclusion Criteria

The inclusion criteria were as follows: (1) age range 45–75 years old; (2) meets the diagnostic criteria for ischemic stroke, and the onset time to admission is within 7 days; (3) first onset of AIS patients; (4) have had ischemic etiology confirmed by brain computed tomography (CT) or MRI scan; (5) National Institutes of Health Stroke Scale (NIHSS) score ranges from 4 to 8 points; (6) subcortical lesions, not involving the cortex; and (7) awake, alert, and capable of participating in research.

### 2.3. Exclusion Criteria

The exclusion criteria were as follows: (1) recurrent stroke and cerebral hemorrhage identified based on clinical history and MRI examination; (2) other abnormal brain lesions discovered by cranial MRI; (3) post‐stroke aphasia, thrombolysis, thrombectomy, and bridging therapy; (4) having a history of other mental or neurological disorders; (5) patients with severe respiratory, circulatory system diseases, and liver and kidney diseases that pose a serious threat to their lives; (6) those who have been lost to follow‐up or refused follow‐up for more than 3 months; (7) patients with intolerance or contraindications to MRI; and (8) MRI scanning included fear of noise or low‐temperature environments, diagnosis of spatial claustrophobia and metal implants.

### 2.4. Handling of Adverse Events

During the treatment process, the doctor should explain the treatment plan to the patient in detail and request honest feedback regarding any changes in their condition after treatment, avoiding any leading questions. Additionally, while observing clinical efficacy, it is important to monitor the patient for adverse reactions. In acupuncture therapy, close attention should be paid to the occurrence of needle fainting, bent needles, stuck needles, or subcutaneous hematomas, and patients should be instructed to maintain a proper position and remain relaxed.

### 2.5. Clinical Assessment

The participants in both groups were assessed by a trained evaluator, who was blind to patients’ assignment, a total of three times: at baseline (on the day of enrollment), week 8 (at the end of the acupuncture intervention), and week 12 (after a 12‐week follow‐up period post‐intervention).

### 2.6. Primary Outcome

The NIHSS is conducted both during the acute phase of stroke onset and throughout the treatment process to assess neurological deficits. It provides a detailed reflection of the progression of the condition, including whether it has worsened or improved. The scale mainly includes assessments of consciousness, eye movement, visual field, facial paralysis, upper and lower limb movement, sensation, coordination, language (aphasia and dysarthria), and neglect. The total score is 42 points, with higher scores indicating more severe neurological impairment.

### 2.7. Secondary Outcomes

The Fugl‐Meyer assessment (FMA) is used to evaluate upper and lower limb function before and after treatment in patients. It includes 33 items for upper limb function, with a total score of 66 points, and 17 items for lower limb function, with a total score of 34 points. The higher the score, the better the recovery of the patient’s limb function. This scale is used to assess the motor function in hemiplegic patients of all ages after a stroke. It is applicable in both clinical and research settings to determine the severity of the condition, describe motor recovery, and plan and evaluate the treatment.

### 2.8. MR Image Acquisition

All participants underwent high‐resolution *T*_1_‐weighted and rs‐fMRI scans on the 3.0 T MR scanner (UMR 770, Shanghai United Imaging Healthcare Co. Ltd, China). The acquisition parameters for *T*_1_‐weighted images (*T*_1_WI) were as follows: 160 sagittal slices, repetition time (TR) = 7.3 ms, echo time (TE) = 3.1 ms, slice thickness = 1 mm, field of view (FOV) = 256 × 256 mm^2^, and voxel size = 1 × 1 × 1 mm^3^. The rs‐fMRI data were acquired with the following parameters: 36 axial slices, TR = 2 s, TE = 30 ms, slice thickness = 3.5 mm, FOV = 230 × 230 mm^2^, voxel size = 3.5 × 3.5 × 3.5 mm^3^, and 220 volumes. Images were acquired at baseline, week 8, and week 12, respectively.

### 2.9. Structural Magnetic Resonance Image Preprocessing

*T*_1_‐weighted images were first bias‐corrected and then segmented into the GM, white matter, and cerebrospinal fluid. Next, the nonlinear modulation of GM maps was aligned using the diffeomorphic anatomical registration through the exponential lie algebra template and spatially normalized into the Montreal Neurological Institute (MNI) space with a resolution of 1.5 × 1.5 × 1.5 mm^3^. Subsequently, the normalized GM maps were smoothed using a Gaussian kernel with an 8‐mm full width at half maximum (FWHM). GMV data were registered to a symmetric surface template, enabling the flipping of lesions to the contralateral hemisphere [14, 15]. For patients with a right‐sided lesion, both lesion and cortical thickness data were flipped to ensure that the left hemisphere consistently represented the lesion side, in accordance with common practice in stroke literature [15–17]. Using Python software, flip the imaging data with right‐sided lesions to the left side, ensuring the lesions are located in the same position on the left. This helps to avoid potential lateralization effects and facilitates overall analysis. Structural images were spatially normalized to the MNI template without lesion masks.

### 2.10. Resting‐State Functional Magnetic Resonance Image Preprocessing

The rs‐fMRI data processing and analyses were conducted using the DPARSF 5.1 toolbox in SPM12, which is based on MATLAB 2020 b. The preprocessing pipeline included slice timing correction, within‐subject EPI image realignment, and spatial normalization of the fMRI images to the MNI standard space using *T*_1_‐weighted images with an EPI template. Following normalization, EPI images were resampled into 3 × 3 × 3 mm^3^ voxels and smoothed with a 6‐mm FWHM Gaussian kernel. Finally, the images were subjected to linear detrending and temporal bandpass filtering (0.01–0.08 Hz) to attenuate low‐frequency drifts and physiological noise.

During MRI preprocessing, rigid‐body realignment was performed to correct for head motion. Participants with head motion greater than 3° in rotation or excessive translation were excluded from further analysis.

### 2.11. Seed‐Based FC Analysis

Each brain region exhibiting statistically significant GMV differences was defined as a seed region of interest (ROI), and further FC analysis was performed in both the AWT and WT groups. For each patient, across different time points, Pearson correlation coefficients were calculated between the mean time series extracted from the same seed ROI and the time series of all other voxels in the brain, generating FC maps. Subsequently, Fisher’s *r*‐to‐*z* transformation was applied to convert the FC maps into *z*‐score maps, improving the normality of the correlation coefficients.

### 2.12. Statistical Analysis

Analysis of the continuous variable data involved conducting independent sample *t*‐tests or Mann‐Whitney *U* tests, while categorical data were compared using either a chi‐square test or Fisher’s exact test. A two‐sample test was employed to investigate the alterations in GMV and FC in two groups of AIS patients and to assess the impact of acupuncture on GMV in the cerebral cortex. All imaging analyses were performed blinded to group allocation and clinical information to avoid assessment bias. Multiple comparison corrections were applied using Gaussian random field (GRF) correction correction for all whole‐brain GMV and FC analyses to control type I error. The statistical significance threshold was set to *p*  < 0.001 based on voxel level and *p*  < 0.05 based on cluster level. The voxel <10 was not included in the study, and the two‐sided test was set as the significance level. Spearman’s correlation coefficient was utilized to correlate the ROI values extracted from the positive brain regions with the NIHSS and FMA.

Owing to considerable participant attrition during follow‐up, only 17 patients completed all three time‐point assessments. The small number of longitudinal observations and potential nonrandom missing data precluded the use of linear mixed‐effects models or repeated‐measures ANOVA. Therefore, we analyzed the data at each time point separately and interpreted the long‐term findings with caution.

## 3. Results

### 3.1. Demographic and Clinical Information

**Figure 1:**
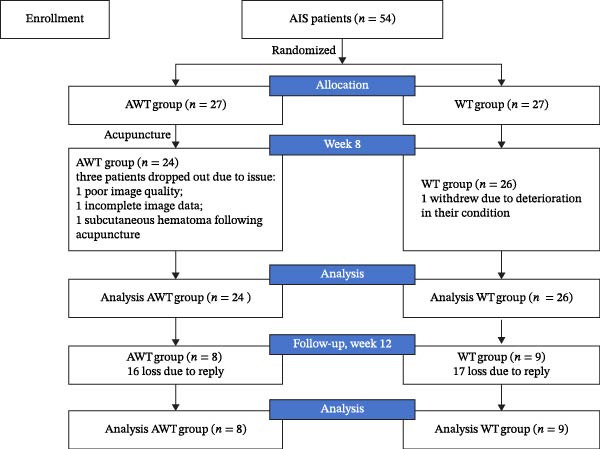
The flowchart of a clinical study. AIS, acute ischemic stroke; AWT, acupuncture combined with western medicine treatment; WT, western medicine treatment.

**Figure 2:**
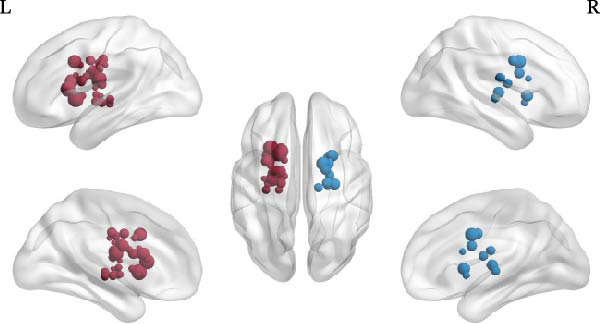
Lesion distribution map of AIS patients. Red sphere: lesions were located on the left side; blue sphere: lesions were located on the right side. The number of spheres represents the number of patients, *n* = 50 cases. The size of the spheres represents the volume of the lesion; each sphere represents a different location of AIS patient.

**Figure 3:**
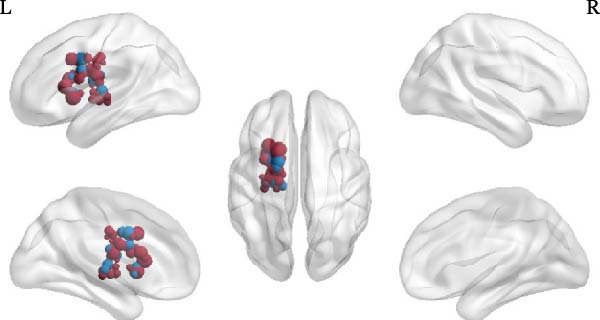
Flipped lesion mapping distribution map of AIS patients.

Demographic and clinical characteristics of the participants are presented in Tables 1 and 2 and Figure 1. A total of 54 patients with AIS were initially enrolled at baseline. Several participants were subsequently excluded for various reasons. Specifically, three patients in the AWT group dropped out due to poor image quality, incomplete imaging data, or subcutaneous hematoma after acupuncture, leaving 24 patients in the AWT group. In the WT group, one patient withdrew at week 8 owing to clinical deterioration, resulting in 26 patients remaining in the WT group. Ultimately, 50 AIS patients were included in the final analysis. Following the completion of acupuncture intervention, all participants were continuously followed up for 12 weeks. Only 17 patients completed the 12‐week follow‐up, including 8 from the AWT group and 9 from the WT group. The mean age was 62.71 ± 9.03 years in the AWT group, with 16 males, whereas the WT group had a mean age of 61.04 ± 7.09 years and 11 males. All patients were in the acute stroke stage with subcortical lesions. Among these patients, 38 had left‐sided lesions and 12 had right‐sided lesions. Imaging data of right‐lesion participants were flipped to the left hemisphere using Python software to standardize all lesions to the left side (Figures 2 and 3). No significant differences were found between the two groups in terms of age, gender distribution, history of hypertension, type 2 diabetes mellitus, hyperlipidemia, coronary heart disease, lesion volume, NIHSS score, FMA score, smoking history, or alcohol consumption history.

**Table 1:** Demographic and clinical information of participants at the week 8.

Variable	AWT (*n* = 24)	WT (*n* = 26)	*F/*χ^2^/*Z*	*p*
Age (year), mean ± SD	62.71 ± 9.03	61.04 ± 7.09	2.157	0.469^a^
Gender (male/female)	16/8	11/15	2.981	0.084^b^
Years of education (year)	6.71 ± 3.33	6.35 ± 3.08	0.373	0.691^a^
Hypertension, *n* (%)	16 (66.67)	14 (53.85)	0.855	0.355^b^
Type 2 diabetes, *n* (%)	9 (37.5)	11 (42.31)	0.120	0.729^b^
Coronary heart disease, *n* (%)	6 (25)	8 (30.77)	0.206	0.650^b^
Hyperlipidemia, *n* (%)	5 (20.83)	8 (30.77)	0.640	0.424^b^
Smoking, *n* (%)	11 (45.83)	8 (30.77)	1.202	0.273^b^
Alcohol, *n* (%)	8 (33.33)	6 (23.08)	0.651	0.420^b^
Lesion volume (mL), *M* (IQR)	8.55 (2.2)	7.52 (2.08)	−1.874	0.061^c^

^a^Independent sample *t*‐test.

^b^Chi‐square test.

^c^Nonparametric rank‐sum test.

**Table 2:** Demographic and clinical information of participants at the week 12.

Variable	AWT group (*n* = 8)	WT group (*n* = 9)	*χ^2^*	*p*
Age (year), *M* (IQR)	58.50 (17.50)	65.00 (12.00)	−0.145	0.906^b^
Gender (male/female)	6/2	3/6	—	0.153^a^
Years of education (year)	8.00 (5.25)	6.00 (5.55)	0.843	0.432^b^
Lesion volume (mL), *M* (IQR)	8.07 (3.83)	8.67 (2.99)	−0.770	0.481^b^
NIHSS, *M* (IQR)	2.00 (0.00)	3.00 (1.00)	−3.759	＜0.001^b^
FMA, *M* (IQR)	98.00 (2.50)	95.00 (6.50)	−2.448	0.015^b^

^a^Fisher test.

^b^Nonparametric rank‐sum test.

A total of 54 patients were enrolled at baseline. After 8 weeks of acupuncture intervention, 50 participants completed the follow‐up, with a retention rate of 92.6% and a loss‐to‐follow‐up rate of 7.4%. For the 12‐week follow‐up, data were available for only 17 participants, corresponding to a retention rate of 31.5% (17/54) and a loss‐to‐follow‐up rate of 68.5% (37/54). The primary reasons for loss to follow‐up were difficulties in imaging acquisition and poor image quality, which accounted for 80% of all losses; refusal to participate in follow‐up accounted for 10%, and other reasons accounted for the remaining 10%. These factors collectively resulted in substantial data loss between follow‐up time points. Baseline demographic and clinical characteristics were compared between participants who completed the 12‐week follow‐up and those who were lost to follow‐up. No significant differences were observed between the two groups in terms of age, gender, previous hypertension, type 2 diabetes mellitus, hyperlipidemia, coronary heart disease, lesion volume, NIHSS score, mRS score, FMA score, smoking history, or alcohol consumption history (Table 3).

**Table 3:** Baseline characteristics between follow‐up completers and noncompleters.

Variable	Completers (*n* = 17)	Noncompleters (*n* = 37)	*Z/χ^2^*	*p*
Age (year), *M* (IQR)	60 (14)	59 (12.5)	−0.411	0.681^b^
Gender (male/female)	9/8	20/17	0.086	0.770^a^
Years of education (year)	8 (6)	6 (4.89)	−0.623	0.533^b^
Hypertension, *n* (%)	11 (64.7)	21 (56.76)	0.305	0.581^a^
Type 2 diabetes, *n* (%)	7 (41.18)	15 (40.54)	0.725	0.394^a^
Coronary heart disease, *n* (%)	3 (17.65)	12 (32.43)	1.269	0.260^a^
Hyperlipidemia, *n* (%)	5 (29.41)	12 (32.43)	0.049	0.824^a^
Smoking, *n* (%)	6 (35.29)	16 (43.24)	0.305	0.581^a^
Alcohol, *n* (%)	4 (23.53)	13 (35.14)	0.727	0.394^a^
Lesion volume (mL), *M* (IQR)	8.67 (1.68)	7.44 (2.13)	−1.802	0.071^b^
NIHSS, *M* (IQR)	5 (1)	5 (2)	−0.986	0.324^b^
mRS, *M* (IQR)	3 (1)	3 (1)	−1.118	0.264^b^
FMA, *M* (IQR)	85 (9)	88 (5)	−0.915	0.360^b^

*Note:* Completers: participants who completed the 12‐week follow‐up.

^a^Chi‐square test.

^b^Nonparametric rank‐sum test.

### 3.2. Clinical Efficacy Outcomes

The primary and secondary clinical efficacy outcomes are shown in Table 4.

**Table 4:** Results of the between‐group comparison of clinical efficacy scale.

Variable	AWT group	WT group	*Z*	*p*
NIHSS, *M* (IQR)				
Baseline	5.50 (1.00)	5.00 (1.25)	−1.747	0.084^a^
Week 8	3.00 (1.00) ^∗∗∗^	3.50 (1.25) ^∗∗∗^	−2.140	0.036^a^
Week 12	2.00 (0.00) ^∗∗∗^^c^	3.00 (1.00) ^∗∗∗^^b^	−5.953	＜0.001^a^
FMA, *M* (IQR)				
Baseline	85.50 (6.00)	86.50 (5.00)	−0.917	0.359^a^
Week 8	93.00 (5.00) ^∗∗∗^	90.00 (1.00) ^∗∗^	−3.399	＜0.001^a^
Week 12	98.00 (3.00) ^∗∗∗^^c^	95.00 (5.00) ^∗∗^^c^	−4.298	＜0.001^a^

^a^Non‐parametric rank‐sum test for intergroup comparison between the two groups during the same period. Week 8: 8 weeks of acupuncture intervention. Week 12: 12 weeks of follow‐up after the completion of acupuncture intervention. Nonparametric tests were conducted comparing the scores at week 8 and 12 with baseline, revealing statistically significant differences.

^b^No significant difference compared to the 8‐week post‐enrollment assessment (*p* > 0.05).

^c^Comparison between the week 12 and week 8 assessments, *p* < 0.01.

^∗^ *p* < 0.05.

^∗∗^ *p* < 0.01.

^∗∗∗^ *p* < 0.001.

In the between‐group analysis, both the AWT and WT groups showed significant reductions in NIHSS scores (*p* < 0.001 for both) and increases in FMA scores (AWT: *p*  < 0.001, WT: *p*  < 0.01) at both week 8 and week 12 compared to baseline. Both groups demonstrated continuous improvement in FMA scores by week 12. However, the AWT group exhibited further reductions in NIHSS scores at week 12 compared to week 8 (*p*  < 0.001), while the WT group showed no significant changes during this period.

### 3.3. Changes in GMV in Patients With AIS Patients After Acupuncture

**Figure 4:**
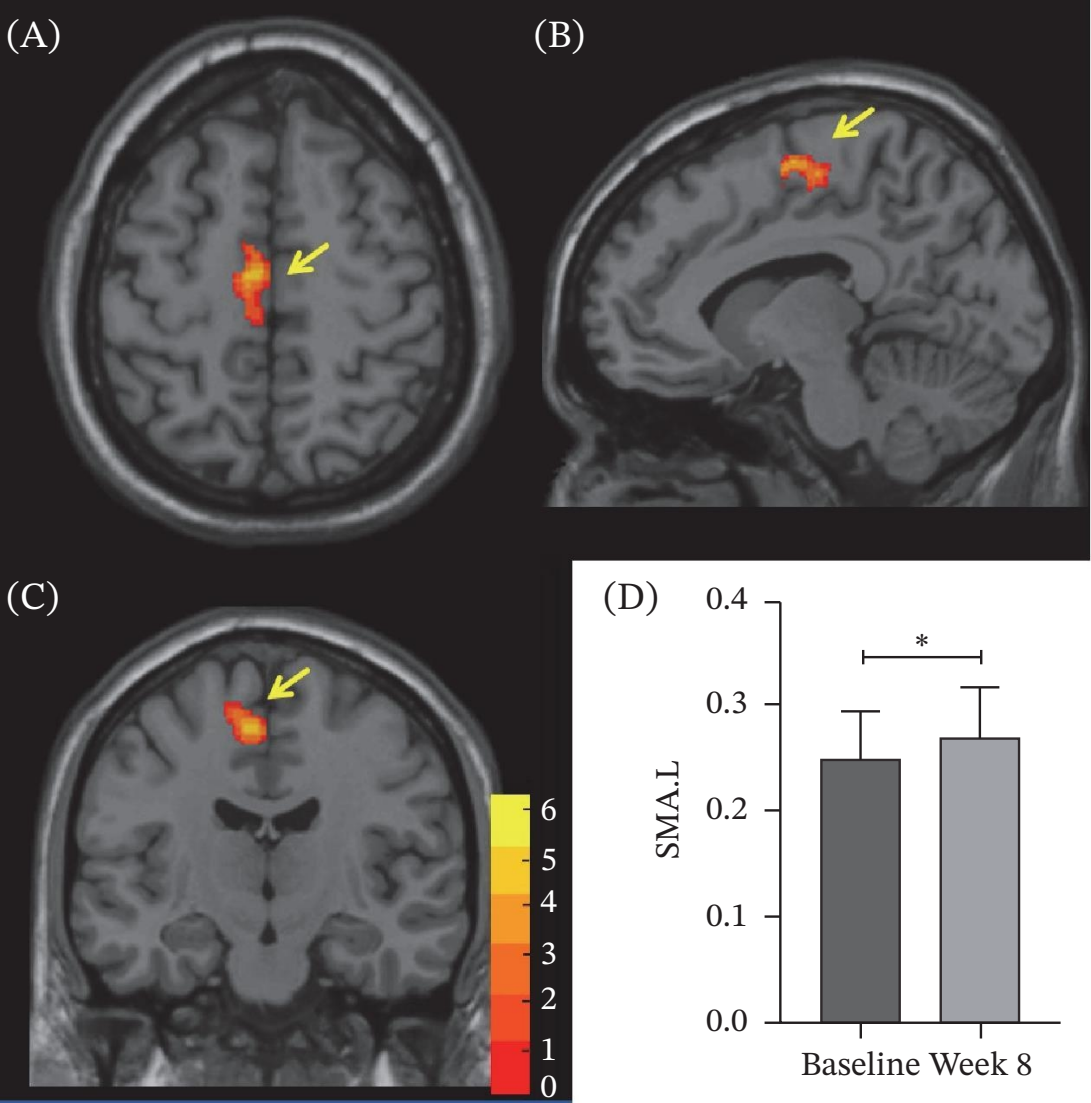
Results of the within‐group comparison of GMV in the AWT group at week 8 (SMA.L). The red mass indicated by the yellow arrow represents the SMA.L, the ipsilesional supplementary motor area. (A) Axial view, (B) sagittal view, (C) coronal view, and (D) results of ROI analysis for the SMA.L before and after acupuncture treatment.  ^∗^ *p*  < 0.05.

**Figure 5:**
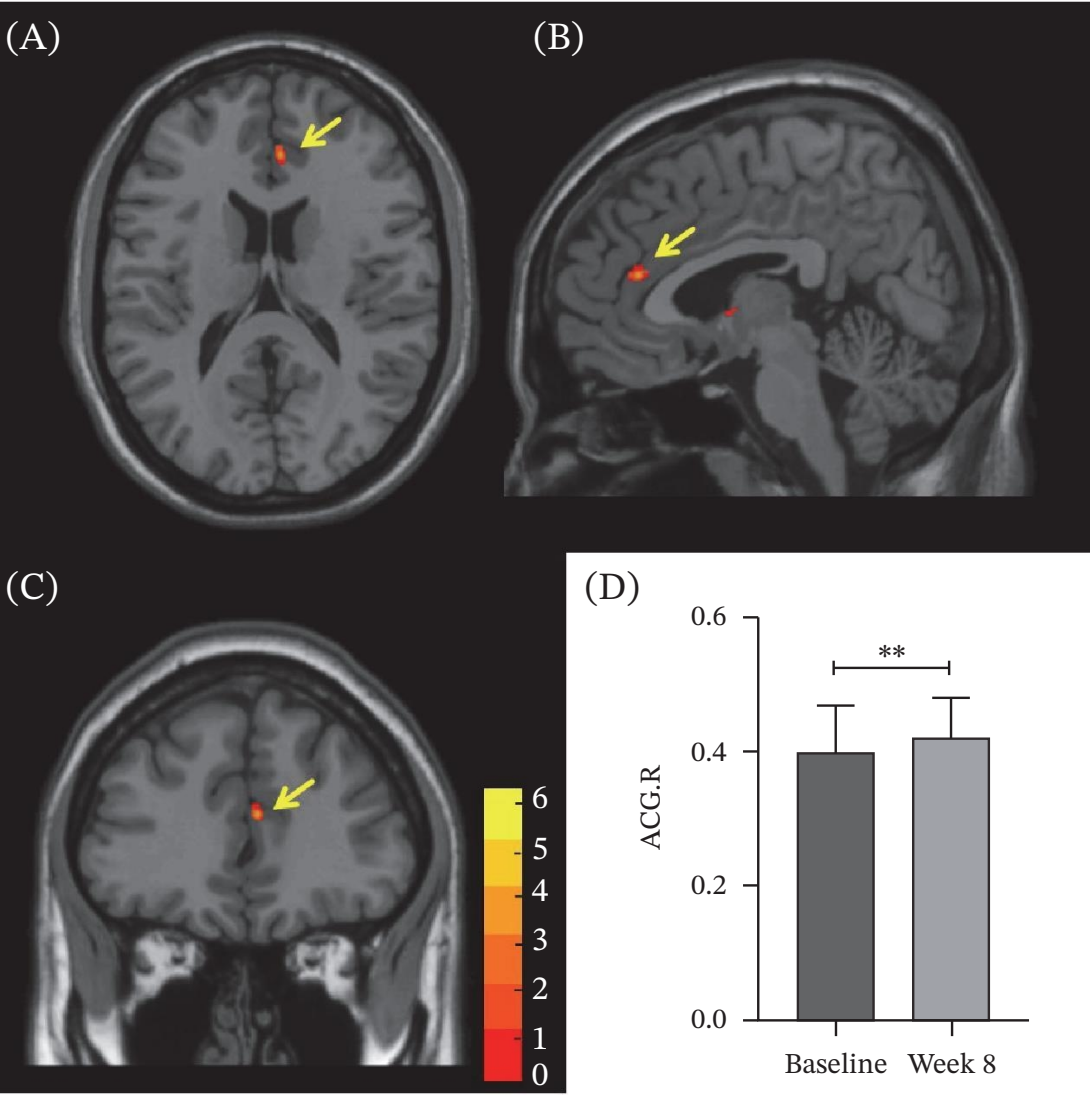
Results of the within‐group comparison of GMV in the AWT group at week 8 (ACG.R). The red mass indicated by the yellow arrow represents the ACG.R, the contralesional anterior cingulate gyrus. (A) Axial view, (B) sagittal view, (C) coronal view, and (D) results of ROI analysis for the ACG.R before and after acupuncture treatment. 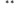 *p* < 0.01.

**Figure 6:**
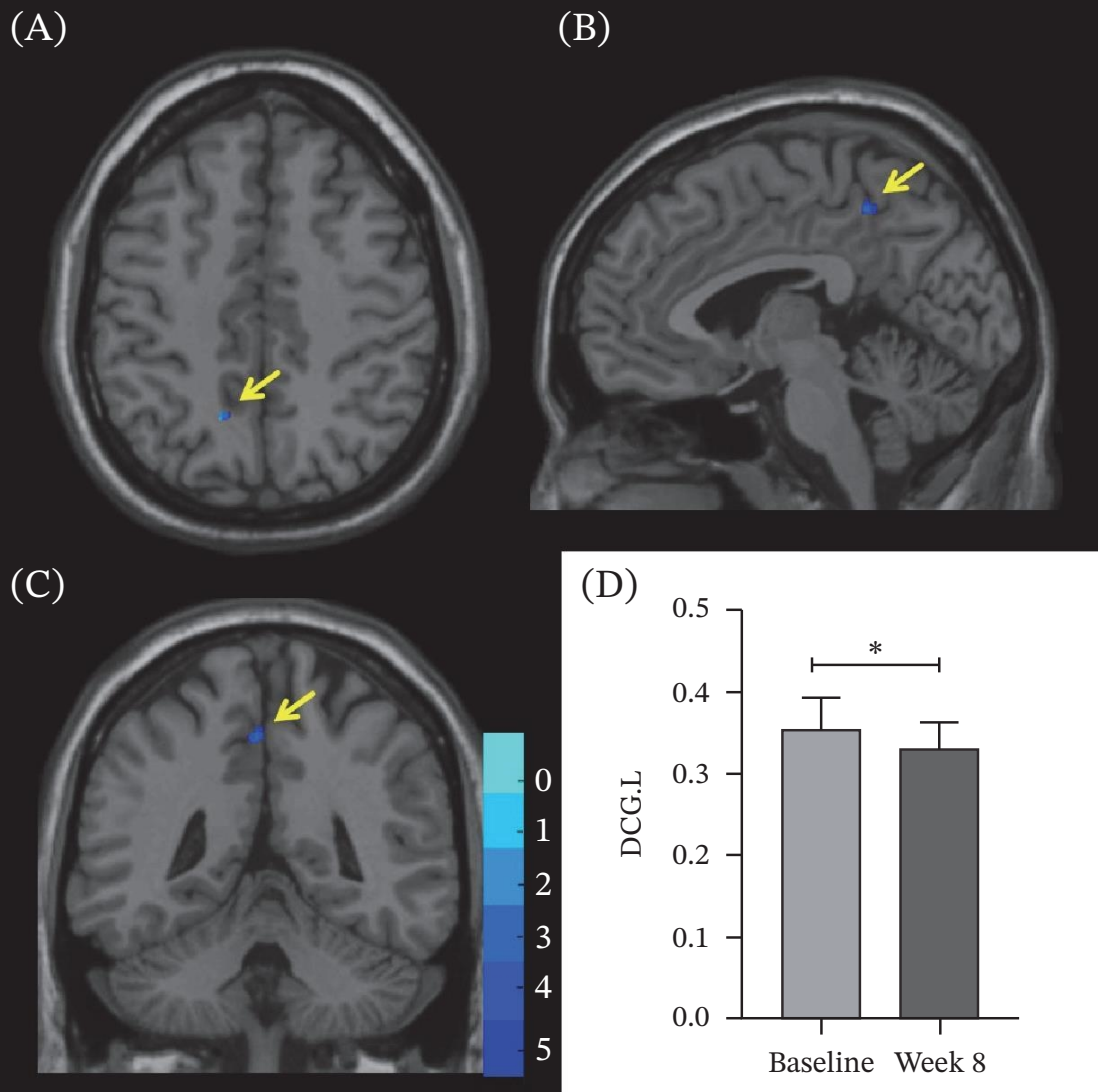
Results of the within‐group comparison of GMV in the WT group at week 8 (DCG.L). The blue mass indicated by the yellow arrow represents the DCG.L, the ipsilesional median cingulate and paracingulate gyrus. (A) Axial view, (B) sagittal view, (C) coronal view, and (D) results of ROI analysis for the DCG.L before and after western medicine treatment.  ^∗^ *p* < 0.05.

**Figure 7:**
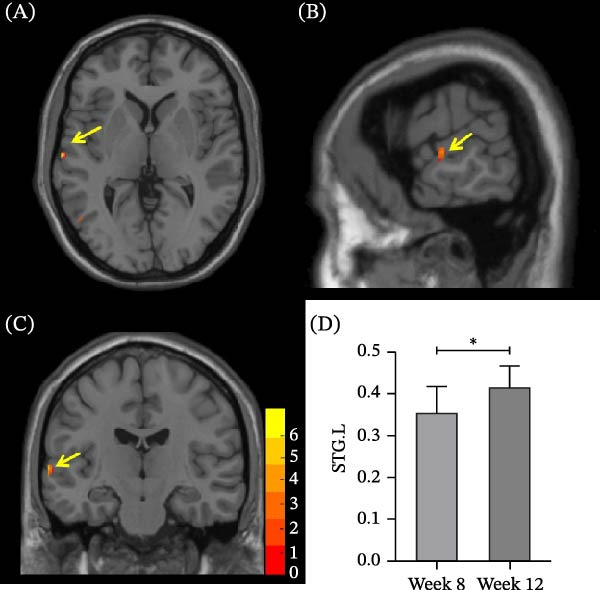
Results of the within‐group comparison of GMV in the AWT group at week 12 (STG.L). The red mass indicated by the yellow arrow represents the STG.L, the ipsilesional superior temporal gyrus. (A) Axial view, (B) sagittal view, (C) coronal view, and (D) results of ROI analysis for the STG.L.  ^∗^ *p* < 0.05.

Compared to baseline, week 8 after acupuncture intervention, the AWT group showed an increase in GMV in the ipsilesional supplementary motor area (SMA; *p* = 0.03) and the contralesional anterior cingulate gyrus (ACG; *p* = 0.007) (Table 5 and Figures 4 and 5). Conversely, the WT group showed a significant decrease in GMV in the ipsilesional median cingulate and paracingulate gyrus (DCG; *p* = 0.013) (Table 6 and Figure 6). Furthermore, at the week 12 follow‐up, the AWT group showed a significant increase in GMV in the ipsilesional superior temporal gyrus (STG) compared to the week 8 (*p* = 0.015) (Table 7 and Figure 7).

**Table 5:** Results of the within‐group comparison of GMV in the AWT group at week 8.

Brain regions	Peak MNI			Voxel	*T*	*p*
	*X*	*Y*	*Z*			
SMA.L	−9	−9	61.5	541	5.2861	0.03
ACG.R	4.5	37.5	16.5	40	5.1858	0.007

*Note*: SMA.L, ipsilesional supplementary motor area; ACG.R, contralesional anterior cingulate and paracingulate gyrus; *X*, *Y*, *Z*, representing the left–right, front‐back, and up‐down directions.

Abbreviation: MNI, Montreal Neurological Institute.

**Table 6:** Results of the within‐group comparison of GMV in the WT group at week 8.

Brain regions	Peak MNI			Voxel	*T*	*p*
	*X*	*Y*	*Z*			
DCG.L	−3	−43.5	51	34	4.7954	0.013

*Note:* DCG.L, ipsilesional median cingulate and paracingulate gyrus.

**Table 7:** Results of the within‐group comparison of GMV in the AWT group at week 12.

Brain regions	Peak MNI			Voxel	*T*	*p*
	*X*	*Y*	*Z*			
STG.L	−66	−16.5	3	19	7.6924	0.015

*Note:* STG.L, ipsilesional superior temporal gyrus.

**Figure 8:**
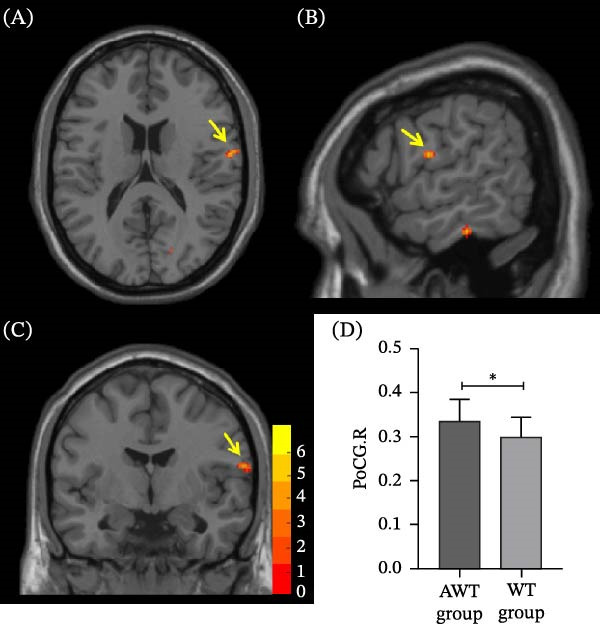
Results of the between‐group comparison of GMV at week 8 (PoCG.R). The red mass indicated by the yellow arrow represents the PoCG.R, the contralesional postcentral gyrus. (A) Axial view, (B) sagittal view, (C) coronal view, and (D) results of ROI analysis for the PoCG.R.  ^∗^ *p* < 0.05.

**Figure 9:**
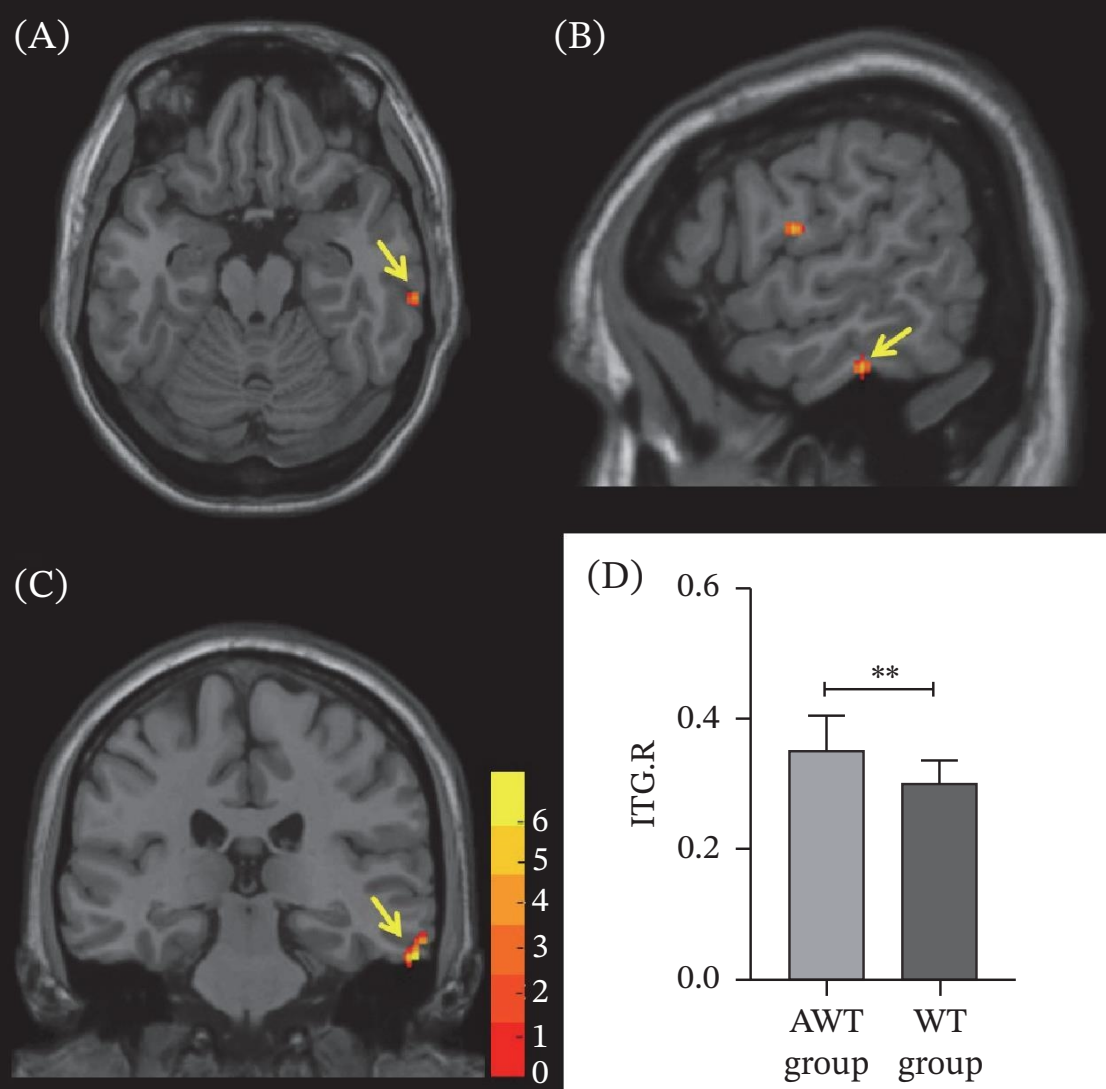
Results of the between‐group comparison of GMV at week 8 (ITG.R). The red mass indicated by the yellow arrow represents the ITG.R, contralesional inferior temporal gyrus. (A) Axial view, (B) sagittal view, (C) coronal view, and (D) results of ROI analysis for the ITG.R.  ^∗∗^ *p* < 0.01.

**Figure 10:**
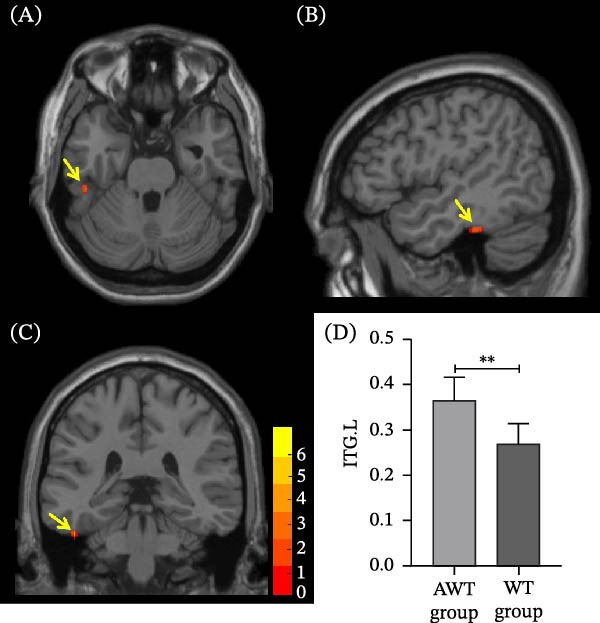
The comparison results between‐group at week 12 (ITG.L). The red mass indicated by the yellow arrow represents the ITG.L, ipsilesional inferior temporal gyrus. (A) Axial view, (B) sagittal view, (C) coronal view, and (D) results of ROI analysis for the ITG.L.  ^∗∗^ *p* < 0.01.

At week 8, between‐group comparisons showed that the AWT group presented significantly greater GMV in the contralesional postcentral gyrus (PoCG; *p* = 0.03) and contralesional inferior temporal gyrus (ITG; *p* = 0.005) relative to the WT group, with all differences reaching statistical significance at *p*  < 0.05 (Table 8 and Figures 8 and 9). At week 12, the AWT group demonstrated a significant increase in GMV in the ipsilesional ITG compared to the WT group (*p* = 0.002) (Table 9 and Figure 10).

**Table 8:** Results of the between‐group comparison of GMV at week 8.

Brain regions	Peak MNI			Voxel	*T*	*p*
	*X*	*Y*	*Z*			
PoCG.R	−9	−9	61.5	51	3.7922	0.03
ITG.R	4.5	37.5	16.5	54	3.9497	0.005

*Note:* PoCG.R, contralesional postcentral gyrus; ITG.R, contralesional inferior temporal gyrus.

**Table 9:** Results of the between‐group comparison of GMV at week 12.

Brain regions	Peak MNI			Voxel	*T*	*p*
	*X*	*Y*	*Z*			
ITG.L	−48	−33	−30	24	4.9986	0.002

*Note:* ITG.L, ipsilesional inferior temporal gyrus.

### 3.4. Changes in FC in Patients With AIS Patients After Acupuncture

**Figure 11:**
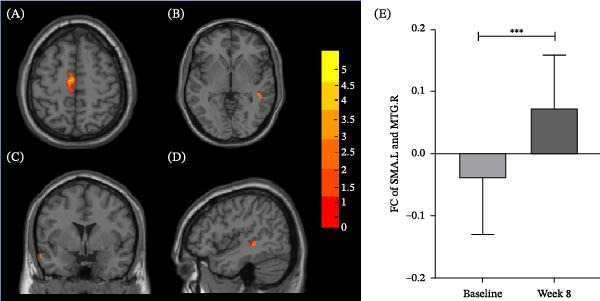
Results of the within‐group comparison of FC in the AWT group at week 8 (MTG.R). Functional connectivity (FC) analysis of the whole brain was conducted using the SMA.L as a seed point. It was found that the FC between the SMA.L and the MTG.R was enhanced. (A) It shows seed point of the SMA.L. The contralateral MTG: (B) it shows the axial view, (C) it shows the coronal view, and (D) it shows the sagittal view. (E) It shows the results of the group‐level FC comparison in the AWT group. 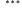 *p* < 0.001.

**Figure 12:**
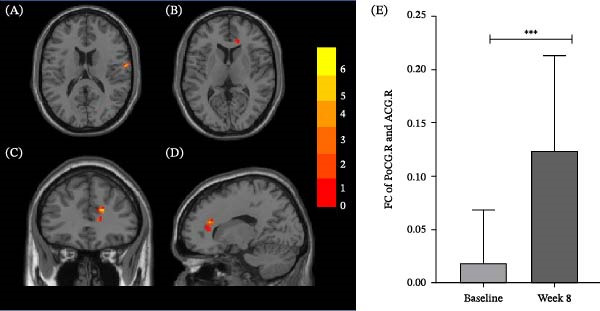
Results of the within‐group comparison of FC in the AWT group at week 8 (ACG.R). FC analysis of the whole brain was conducted using the PoCG.R as a seed point. It was found that the FC between the PoCG.R and the ACG.R was enhanced. (A) It shows the seed point of the PoCG.R. The ACG.R: (B) it shows the axial view, (C) it shows the coronal view, and (D) it shows the sagittal view. (E) It shows the results of the group‐level FC comparison in the AWT group. 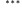 *p* < 0.001.

**Figure 13:**
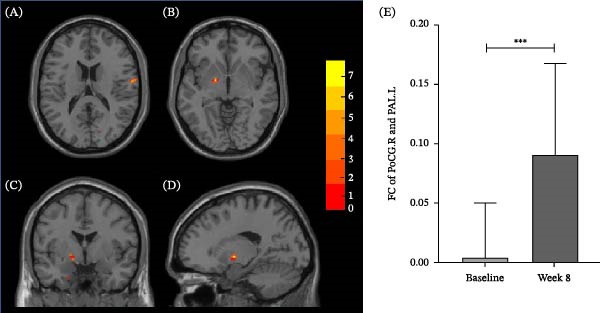
Results of the within‐group comparison of FC in the AWT group at week 8 (PAL.L). Functional connectivity (FC) analysis of the whole brain was conducted using the PoCG.R as a seed point. It was found that the FC between the PoCG.R and the PAL.L was enhanced. (A) It shows the seed point of the PoCG.R. The PAL.L: (B) it shows the axial view, (C) it shows the coronal view, and (D) it shows the sagittal view. (E) It shows the results of the group‐level FC comparison in the AWT group. 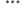 *p* < 0.001.

**Figure 14:**
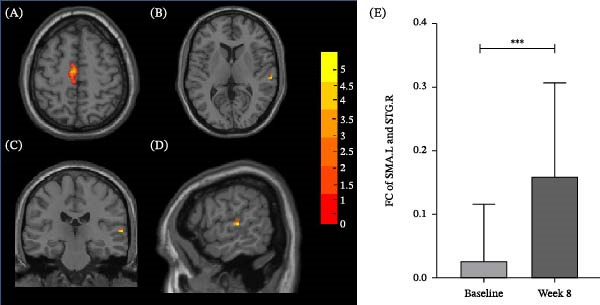
Results of the within‐group comparison of FC in the WT group at week 8 (STG.R). FC analysis of the whole brain was conducted using the SMA.L as a seed point. It was found that the FC between the SMA.L and the STG.R was enhanced. (A) It shows the seed point of the SMA.L. The STG.R: (B) it shows the axial view, (C) it shows the coronal view, and (D) it shows the sagittal view. (E) It shows the results of the group‐level FC comparison in the WT group. 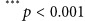.

**Figure 15:**
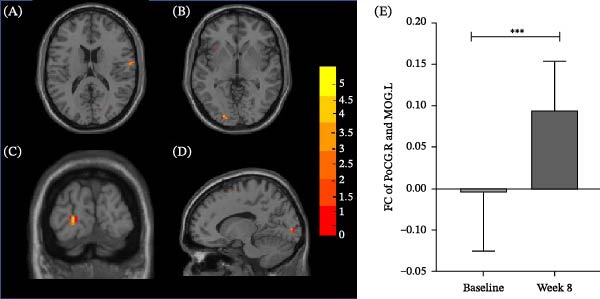
Results of the within‐group comparison of FC in the WT group at week 8 (MOG.L). FC analysis of the whole brain was conducted using the PoCG.R as a seed point. It was found that the FC between the PoCG and the MOG.L was enhanced. (A) It shows the seed point of the PoCG.R. The MOG.L: (B) it shows the axial view, (C) it shows the coronal view, and (D) it shows the sagittal view. (E) It shows the results of the group‐level FC comparison in the WT group. 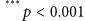.

The seven brain regions exhibiting significant differences in GMV, including the ipsilesional SMA, DCG, STG, and ITG, as well as the contralesional ACG, PoCG, and IT, were used as seed regions for whole‐brain FC analysis. Compared to baseline measurements, inter‐group comparisons revealed that the AWT group showed significantly increased FC between the ipsilesional SMA and the contralesional middle temporal gyrus (MTG; *p*  < 0.001) (Table 10 and Figure 11), between the contralesional PoCG and ACG (*p* < 0.001) (Table 10 and Figure 12), as well as between the contralesional PoCG and the ipsilesional pallidum (PAL; *p* < 0.001) at week 8 (Table 10 and Figure 13). In the WT group, there was an increase in FC between the ipsilesional SMA and the contralesional STG (*p*  < 0.001) (Table 11 and Figure 14), and an increase in FC between the contralesional PoCG and the ipsilesional middle occipital gyrus (MOG; (*p* < 0.001) (Table 11 and Figure 15). Between‐group comparisons showed that no positive FC regions were identified at either week 8 or week 12 after enrollment.

**Table 10:** Results of the within‐group comparison of FC in the AWT group at week 8.

Brain regions	Peak MNI			Voxel	*T*	*P*
	*X*	*Y*	*Z*			
MTG.R	48	−39	−3	10	5.4117	<0.001
ACG.R	15	33	21	25	6.8146	<0.001
PAL.L	−18	−3	−3	20	7.6954	<0.001

*Note:* MTG.R, contralesional middle temporal gyrus; ACG.R, contralesional anterior cingulate gyrus; PAL.L, ipsilesional pallidum; *X*, *Y*, *Z*, representing the left‐right, front‐back, and up‐down directions.

**Table 11:** Results of the within‐group comparison of FC in the WT group at week 8.

Brain regions	Peak MNI			Voxel	*T*	*p*
	*X*	*Y*	*Z*			
STG.R	63	−24	6	12	5.2595	＜0.001
MOG.L	−18	93	0	27	5.1898	＜0.001

*Note:* STG.R, contralesional superior temporal gyrus; MOG.L, ipsilesional middle occipital gyrus.

### 3.5. Spearman Correlation Analysis

**Figure 16:**
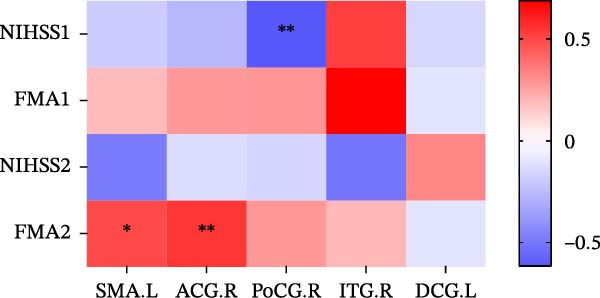
Correlation between GMV and clinical efficacy score at 8 weeks after enrollment was analyzed. NIHSS1: the NIHSS score at 8 weeks post‐enrollment; NIHSS2: the NIHSS score at 12 weeks post‐enrollment. FMA1: the FMA score at 8 weeks post‐enrollment and FMA2: the FMA score at 12 weeks post‐enrollment. 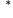 *p* < 0.05; 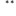 *p* < 0.01.

**Figure 17:**
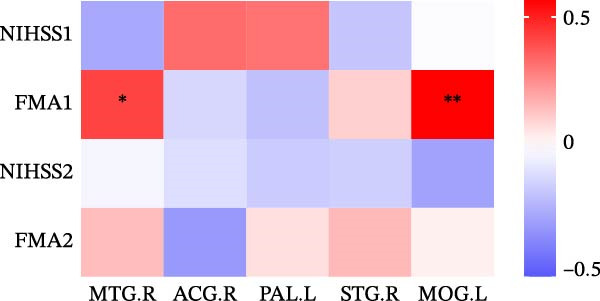
Correlation between FC and clinical efficacy score at 8 weeks after enrollment was analyzed.  ^∗^ indicates *p* < 0.05 and  ^∗∗^ indicates *p* < 0.01.

In the Spearman correlation analysis, a positive correlation was found in the AWT group between the GMV of the ipsilesional SMA at weeks 8 post‐enrollment and the FMA score at week 12 (*p* = 0.016, *R* = 0.487) (Figure 16). Additionally, the GMV in the contralesional ACG showed a positive correlation with the FMA score at week 12 (*p* = 0.006, *R* = 0.540) (Figure 16). Additionally, a negative correlation was also found in the WT group between the GMV of contralesional PoCG and the NIHSS score at week 8 (*p* = 0.004, *R* = −0.540) (Figure 16).The enhanced FC between the ipsilesional SMA and the contralesional MTG in the AWT group was positively correlated with the post‐acupuncture FMA score (*p* = 0.043, *R* = 0.417) (Figure 17). In the WT group, the enhanced FC between the contralesional PoCG and the ipsilesional MOG was positively correlated with the FMA score (*p* = 0.003, *R* = 0.567) (Figure 17).

## 4. Discussion

In the present study, multiple analytical approaches were applied to investigate acupuncture‐induced structural and functional alterations in the cerebral cortex among patients with AIS. Longitudinal analyses revealed widespread GMV changes across both ipsilesional and contralesional hemispheres. At week 8, the AWT group exhibited significantly increased GMV in the ipsilesional SMA and contralesional ACG. In contrast, the WT group showed a notable reduction in GMV within the ipsilesional DCG. Furthermore, at week 12 follow‐up, the AWT group showed a significant increase in GMV in the ipsilesional STG. Using these clusters derived from the longitudinal GMV analyses as seed regions, we further analyzed the longitudinal FC changes and found a significant increase of FC between the ipsilesional SMA as seed brain region and the contralesional MTG, between the contralesional PoCG as seed brain region and contralesional ACG and the ipsilesional PAL at week 8 after acupuncture in the AWT group. In the WT group, there was an increase in FC between the ipsilesional SMA as seed brain region and the contralesional STG and between the contralesional PoCG as seed brain region and the ipsilesional MOG at week 8 in the WT group.

### 4.1. Acupuncture‐Induced Modulation of GMV

Numerous evidence has indicated that stroke patients undergo extensive cerebral structural reorganization, which is closely accompanied by neuroplastic remodeling [9–12, 18–20]. Previous research has revealed increased GMV in the ipsilesional and contralesional precuneus, contralesional SFG, contralateral IFG and MTG in patients with striatal infarcts and increased GMV in the frontal and temporal regions with complete recovery in patients with stroke [3, 18, 21]. Acupuncture has been shown to have beneficial effects on recovery from ischemic stroke, potentially by modifying the brain structure [13, 14, 16]. In our study, the AWT group exhibited increased GMV in the ipsilesional SMA and contralesional ACG following acupuncture intervention, which aligns well with the previous evidence. Extended follow‐up to week 12 further revealed that the AWT group showed significant GMV increases in the ipsilesional STG relative to week 8 baseline assessments. In the WT group receiving no acupuncture intervention, progressive GMV reduction persisted in the ipsilesional DCG after week 8. By comparison, the AWT group displayed elevated GMV in multiple contralesional regions, including the SMA and PoCG. These findings suggest that GM atrophy gradually progresses over time in patients with AIS under conventional treatment alone, whereas acupuncture intervention may attenuate such progressive GM loss, consistent with prior relevant studies. Importantly, persistent GM impairment remained evident in the conventional treatment group at week 8, yet no further deterioration was detected by week 12. This phenomenon may be associated with the small sample size or with compensatory mechanisms in either the ipsilesional or contralesional brain regions. However, due to the small sample size included, this is only a preliminary finding. In order to investigate in more depth whether GM damage persists or changes during the 3 months following the end of acupuncture treatment, subsequent studies will expand the sample size with the aim of more comprehensively evaluating the changes in GMV in the brain regions of patients in the conventional treatment group.

### 4.2. Seed‐Based Whole‐Brain FC

Using these clusters derived from the longitudinal GMV analyses as seed regions, we further analyzed the longitudinal changes in FC. Specifically, in the AWT group at week 8 after acupuncture intervention, we observed a significant increase in FC between the ipsilesional SMA (seed region) and the contralesional MTG, as well as between the contralesional PoCG (seed region) and both the contralesional ACG and ipsilesional PAL. In contrast, in the WT group at week 8, increased FC was detected between the ipsilesional SMA (seed region) and contralesional STG and between the contralesional PoCG (seed region) and ipsilesional MOG. Additionally, acupuncture stimulation modulates bilateral brain regions, as reflected by changes in ReHo and FC, which may be associated with motor recovery following stroke [22–24]. Stroke can lead to a reduction in connectivity between the damaged brain regions and the surrounding areas, thereby affecting motor, sensory, and cognitive functions, resulting in impairments in patients’ limb movement, sensation, and cognition. For example, a study by Chu et al. [25] found that cerebral infarction can lead to the reorganization of functional networks in related brain regions distant from the lesion, resulting in changes in FC. In a clinical study, Ning et al. [10] found that acupuncture at Yanglingquan acupoint could promote FC enhancement in the sensory–motor network areas of the right precentral gyrus, STG, inferior frontal gyrus, as well as left MTG, MOG, and STG. Additionally, FC was also enhanced in the dorsal attention network areas, such as the right precuneus, STG, MTG, and MOG, along with the left cingulate gyrus, posterior cingulate gyrus, and precuneus. Wei et al. [20] enrolled 22 patients with pontine infarcts and found that patients with pontine infarcts favoring the left side of the cerebellum had atrophy of the GMV in the Crus II of the left side of the cerebellum, right lobule VI of the cerebellum, and right earthworm VI in the dynamic followup. Patients with right‐sided infarcts also showed atrophy of GMV in Crus II of the left cerebellum, and analysis of FC using the above abnormal brain areas as seed points revealed that FC between Crus II of the left cerebellum and the left MFG was weakened, and the altered FC was negatively correlated with motor function [20]. Our study also found that acupuncture intervention significantly enhanced FC between the ipsilesional SMA and contralesional MTG, as well as FC strength between the contralesional PoCG and ACG and between the contralesional PoCG and ipsilesional PAL. Accumulated evidence has shown that migraine patients exhibit disrupted FC between the MTG and sensorimotor regions, particularly reduced coupling with the precentral gyrus and PoCG—core regions subserving motor regulation [26, 27]. Another investigation in patients with Moyamoya disease identified aberrant connectivity among the MTG, SMA, and inferior frontal gyrus, supporting the notion that the MTG participates in higher‐order motor planning and sensorimotor coordination [28].

### 4.3. Spearman Correlation Analysis

Correlation analysis indicated that the increase in GMV of the SMA is positively correlated with the FMA scores at week 12. The SMA, as a brain region associated with motor functions within the cerebral cortex, has fiber connections with the primary motor cortex. The FMA primarily assesses the recovery of motor function in patients; therefore, the increase in GMV of the SMA may be associated with the repair of neural cells, increased cerebral blood flow, reduced axonal damage, and decreased neuronal injury following acupuncture. This likely contributes to the early and sustained recovery of motor function in AIS patients within 3 months after the completion of acupuncture intervention [29, 30]. Additionally, the study found that after 8 weeks of acupuncture intervention, there was a corresponding increase in GMV in the ACG region of the lesion‐affected hemisphere, which was significantly positively correlated with FMA outcomes 3 months post‐acupuncture. At week 12 post‐enrollment, a marked increase in GMV was observed in the STG of the affected side in the AWT group compared to week 8. The ACG is part of the limbic system, which is primarily associated with memory, emotion, and cognitive functions; these aspects play a vital role in our daily lives. It is hypothesized that the increase in GMV of the ACG may benefit the recovery of motor function and improve clinical outcomes in AIS patients. Structural remodeling of cognitive‐related cortical areas may contribute to motor recovery, and cognitive strategies may play a beneficial role in the motor recovery of patients with subcortical strokes [18]. This aligns with clinical observations of patients experiencing post‐stroke emotional and cognitive disorders, which often impact the clinical prognosis, corroborating related studies [31]. The STG is mainly involved in language processing, auditory processing, social cognition, and motor‐related cognitive integration, indirectly influencing patients’ neurological function and motor improvements.

This study has confirmed that acupuncture can promote changes in GMV and FC of various brain regions, particularly in the frontal, parietal, temporal, and occipital lobes, which are closely related to multiple neural functions such as movement, sensation, language, and vision, and are commonly affected areas in stroke patients. Acupuncture may activate related GMV and FC regions by compensatory mechanisms on the same side as the lesion or the contralateral side, establishing new or enhancing existing functional connections, thereby bypassing damaged neurons or pathways to achieve neural function compensation, leading to structural and functional remodeling, which aids in the recovery of neural and motor functions in patients. This remodeling of FC and compensatory mechanism may be an important pathway by which acupuncture facilitates rehabilitation in stroke patients.

Furthermore, these findings suggest that acupuncture can serve not only as an adjunctive treatment for stroke rehabilitation but also as part of an integrated therapeutic strategy when combined with traditional therapies such as medication and rehabilitation training. This multifaceted approach may promote the recovery of neural functions and the improvement of motor skills in patients across various dimensions. By integrating multiple therapeutic methods, synergistic effects can be formed within the affected brain regions and their associated networks, maximizing functional recovery and the long‐term remodeling of neural networks. This multimodal, systematic treatment plan will contribute to further optimizing the overall rehabilitation outcomes for stroke patients, providing more reliable evidence for clinical practice and promoting the widespread application of acupuncture in stroke recovery.

## 5. Limitations

There are several limitations in the present study. First, although our follow‐up design enabled the evaluation of the long‐term effects of acupuncture as an adjunct therapy in AIS patients, substantial participant attrition occurred at the 12‐week time point, leading to a relatively small sample size for long‐term outcome analysis. While participant dropout is inevitable in longitudinal neuro‐rehabilitation studies, the reduced sample may limit the statistical power and generalizability of the 12‐week findings. Therefore, the long‐term results should be interpreted with caution. Our preliminary results indicate that acupuncture may provide additional clinical benefits when combined with conventional rehabilitation for AIS patients. Further large‐sample and long‐term follow‐up studies are warranted to validate these observations and explore the potential underlying neurobiological mechanisms of acupuncture. Second, the present study did not strictly restrict the lesion location of enrolled patients. Future investigations should adopt more rigorous inclusion criteria and focus on targeted intervention research stratified by specific infarction sites. Third, consistent with real clinical practice, patients in the control group declined to receive sham acupuncture, which may have introduced a potential bias to the results. Without a sham acupuncture control, it is difficult to separate the specific therapeutic effects of acupuncture from nonspecific influences, including patient expectation, therapeutic attention, and contextual placebo effects. Accordingly, the clinical efficacy reported herein should be interpreted conservatively. Future studies should expand the sample size, strengthen patient communication and education, and improve participant compliance with sham acupuncture protocols, so as to enhance methodological rigor and the reliability of research conclusions.

## 6. Conclusion

This study investigated longitudinal alterations in GMV and FC among patients with AIS receiving acupuncture treatment over a 3‐month follow‐up period. We found that acupuncture was associated with progressive changes in GMV and FC, mainly localized to the contralesional PoCG and SMA. These alterations may correlate with improvements in motor and sensory recovery in the AWT group. The present findings suggest that acupuncture may modulate brain regions involved in motor and sensory functions, which could potentially facilitate post‐stroke neural reorganization and functional recovery.

## Funding

This work was supported by the National Science and Technology Innovation 2030—Major Program of “Brain Science and Brain‐Like Research” (Grant 2022ZD0211800), the Anhui Province Lu’an Science and Technology Plan Project (Grant 2022lakj024), the Research Project of the Teaching Hospital of Wannan Medical College (Grant WK2023JXYY064), and the Research Fund Project Grant of Anhui Medical University (Grant 2023xkj309).

## Conflicts of Interest

The authors declare no conflicts of interest.

## Data Availability

The data that support the findings of this study are available from the corresponding author upon reasonable request.
